# Paving the Way to Innovative, Child-Friendly Pediatric Diagnostic Methods for Tuberculosis: Introduction of Stool-Based Testing in Ukraine

**DOI:** 10.3390/tropicalmed9090209

**Published:** 2024-09-11

**Authors:** Olena Diuzheva, Liudmyla Skoklyuk, Nina Zherebko, Anna Barbova, Myroslava Germanovych, Eveline Klinkenberg, Oleksii Bogdanov, Gunta Dravniece

**Affiliations:** 1 PATH, 25B, Shota Rustaveli St., 01033 Kyiv, Ukraine; odiuzheva@path.org (O.D.); lskoklyuk@path.org (L.S.); nzherebko@path.org (N.Z.); mgermanovych@path.org (M.G.); eveline@connecttb.org (E.K.);; 2 Central Reference Laboratory for Microbiological Diagnostics of Tuberculosis of the Ministry of Health of Ukraine, 10 Amosova St, 03038 Kyiv, Ukraine; annamint0410@gmail.com

**Keywords:** Xpert MTB/RIF Ultra, fecal sample/stool, diagnostic algorithm, pediatric TB, Ukraine, tuberculosis

## Abstract

Like many countries, Ukraine faces challenges with diagnosing tuberculosis (TB) in children due to the paucibacillary nature of the disease and difficulty obtaining respiratory samples. To improve diagnostic efficiency, stool testing is being integrated into routine pediatric TB services. This started with a pilot introduction at 12 regional TB facilities, where stool was collected for children with a preliminary diagnosis of TB, based on clinical and/or radiological or laboratory findings, in addition to routine testing. For 168 children, a stool test was conducted between November 2021 and September 2022, with samples submitted in all 12 pilot regions. For 132 children, other samples were available in addition to stool. *Mycobacterium tuberculosis* (MTB) was bacteriologically confirmed in 37 children (in stool for 18 children). For 7 of the 18 children with MTB in stool, stool was the only sample in which MTB was detected. Rifampicin resistance was detected in seven children (in stool for three). This noninvasive TB diagnostic sample is especially beneficial for young children who cannot produce sputum. Early detection of TB and its drug-resistant strains in children will allow medical workers to provide safer and more effective treatment and save more lives. Based on the pilot implementation, Ukraine’s national TB program began implementing stool testing throughout the country.

## 1. Introduction

Globally, more than half of children estimated to have tuberculosis (TB) are not diagnosed, and among children under 5 years, 58% are missed [1]. Challenges in diagnosing TB disease in children are related to the paucibacillary nature of the disease in children and difficulties in obtaining respiratory samples, especially in children under 5. Children also have a wide range of manifestations and severity of TB, which can resemble other common childhood diseases such as pneumonia. As a result of these challenges, children are often treated for TB empirically based on clinical features, chest X-ray examination, tuberculin skin tests, or a history of contact with a TB patient. This may lead to over- or underdiagnosis of TB in children.

Ukraine, like many other countries, faces challenges with TB diagnosis in children. In Ukraine, the estimated proportion of children with TB is 5% to 10% of all people diagnosed with TB [2]. Recent national data, however, show that the proportion of children with TB among all notified TB patients in the country is approximately 2%, varying by regional (oblast) level [3]. In 2020, the proportion of notified pediatric TB cases varied from 0.5% to 5.8% by region. The proportion of pediatric TB cases that are bacteriologically confirmed is around 20%, varying from 12% to 22% in the last 5 years [4]. In 2020, there were 377 pediatric TB notifications. Ukraine has a high burden of drug-resistant TB (DR-TB), with around 30% of all TB patients affected [2]. In 2020, 115 children started DR-TB treatment [4].

Since 2020, the World Health Organization (WHO) has recommended stool-based testing with GeneXpert as a primary TB diagnostic test for children with symptoms of pulmonary TB [5,6,7]. Stool testing offers advantages for pediatric diagnosis; it does not require an invasive procedure for children who cannot produce sputum, and it does not require specialized skills for healthcare workers [1]. Feasibility has been shown in resource-limited settings; however, gaps remain between recommendations and effective introduction and country-wide scale-up within national TB programs [8,9].

Beginning in 2021, the USAID-funded Support TB Control Efforts in Ukraine (STBCEU) project, implemented by PATH, conducted a pilot implementation in 12 of Ukraine’s 27 regions to introduce stool as an alternative diagnostic specimen. The aim was to improve the efficiency and quality of TB diagnostics in children by expanding use of noninvasive test samples and to develop an algorithm for integrating the testing of such samples into routine pediatric TB services. Here we describe the results and experiences of the pilot implementation of stool testing.

## 2. Materials and Methods

**Figure 1:**
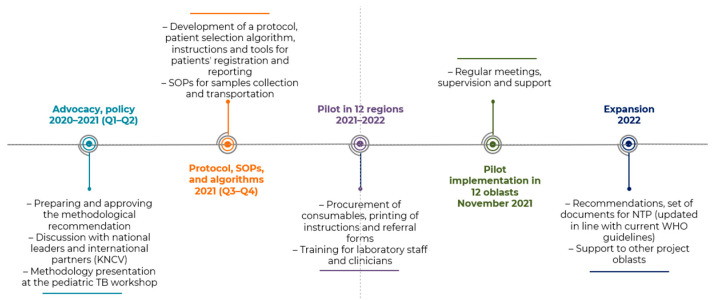
Key milestones in the introduction of stool testing with Xpert^®^ MTB/RIF Ultra.

The pilot implementation (pilot) of stool testing began in November 2021 in 12 regions (Cherkaska, Chernihivska, Dnipropetrovska, Donetska, Khersonska, Kirovohradska, Kyivska, Lvivska, Mykolaivska, Odeska, Poltavska, and Zaporizka). The 12 regions combined cover about half of the population of Ukraine, and include about 21 million people, 16% of them under 15 years of age [10]. Stool testing was introduced alongside other routine testing already conducted for children. The focus during the pilot was on including children with a preliminary diagnosis of TB to demonstrate the role stool could play as a noninvasive sample. Figure 1 provides the timeline and key steps taken in Ukraine to introduce stool testing.

### 2.1. Enabling Policy, Advocacy, and Capacity-Building

As a first step, the national TB reference laboratory, with support from STBCEU, developed, printed, and distributed methodological recommendations on extrapulmonary sample types including stool to all pilot regions. This was needed to ensure stool could be accepted as a sample for TB within the health system. As stool was made part of the routine samples, no specific consent was needed for obtaining stool specimens. Parents did provide verbal consent for any clinical work up or diagnostic test proposed by the TB pediatrician. To generate interest and advocate for stool testing, STBCEU and partners organized a two-day seminar on childhood TB. The project team and key national experts presented the latest WHO recommendations, including the use of stool samples to diagnose TB in children, and discussed the pediatric TB situation in Ukraine. International experts presented additional scientific and practical experience, including new achievements in TB detection and approaches to treat and rehabilitate children with TB. Case study presentations linked theory with practice for healthcare providers across specialty areas. Held on International Children’s Day, the seminar became a key national-level event in the pediatric TB arena, drawing attention to the importance of children’s health and the need for improved diagnostic methods.

To prepare for the pilot, the STBCEU team developed an education and training package using the KNCV Simple One-Step (SOS) Stoolbox [11] and other resources. Given the restrictions for in-person trainings linked to COVID-19, as well as positive experience with the use of pre-recorded video lectures during the pandemic, the STBCEU team developed short video lectures to introduce innovative TB diagnostic methods among children [12]. Combined virtual training was conducted for both clinical and laboratory staff, providing a general introduction to the project and procedures, followed by dedicated seminars for clinical and laboratory staff. Clinical staff were introduced to the use of stool as a specimen to diagnose TB in children, highlighting the benefits of this noninvasive sample to help improve the quality of anti-TB services in pediatric practice, and to the importance of strengthening clinical diagnostics as part of expanding the testing modalities on the Xpert platform. Laboratory staff, who were already competent in performing sputum sample processing and testing on the Xpert platform, were trained in stool processing in an online training wherein the standard operating procedures (SOPs) for stool processing were demonstrated. The project team conducted additional on-the-job training and mentoring for clinical and laboratory staff during the pilot.

### 2.2. Routine TB Investigation and Mycobacterial Diagnostic Testing

In the Ukraine, the routine referral path for diagnosis of TB in children involves several steps. First, an indication of TB is made by a family doctor. Following an initial clinical examination and chest X-ray, patients presumed to have TB are referred by the family doctor to a pediatric TB doctor at the regional TB center. There, the child undergoes further examination starting with a clinical workup and collection of samples. As per the national guidelines, 2 specimens are recommended to be collected for bacteriological testing. The first specimen is collected on the spot at the time of the first patient’s visit and tested using the Xpert MTB/Rif^®^ (Ultra) method. The second specimen is used for culture and drug susceptibility (DST) testing. For collection, the patient is given a container to take home and sputum is to be collected in the morning on an empty stomach. After collection, the sample is to be brought to the facility within 2 h of collection and kept in cold chain as much as possible. If the child is unable to produce a sputum sample, a nasopharyngeal, gastric aspirate, or induced sputum sample is attempted to be collected for bacteriological testing. Based on the clinical evaluation, bacteriological testing, and X-ray results, the pediatric TB doctor makes a TB diagnosis and decision on TB treatment initiation.

For the pilot, stool was added as an alternative sample and pediatric TB doctors were encouraged to collect one stool sample for each child with a preliminary diagnosis of TB. For outpatients, when it was not possible to collect a stool sample from the child at the time of outpatient admission, parents/guardians were asked to collect it at home and deliver it to the laboratory as soon as possible. For inpatients, sample collection was performed by attending nurses.

### 2.3. Sample Processing

Collected samples were sent to the regional laboratory for processing on the Xpert platform. To characterize the consistency of stool, 3 categories were used: formed (hard), unformed (soft), and taking the form of a container (liquid). Processing of the stool was performed using the SOS stool method as described by de Haas et al. [13]. Xpert MTB/RIF Ultra cartridges were used for testing. Samples collected for culture and DST were processed as per routine standard operating procedures and inoculated on both solid (LJ) and liquid (MGIT) media.

### 2.4. Monitoring, Data Management, and Analysis

The TB pediatrician at each regional site shared information with the STBCEU project team monthly on the patients’ clinical exams, samples collected, and test results. In each oblast, the TB pediatrician oversaw and monitored activities and supported implementation by mentoring the TB general doctors and other TB pediatricians of the TB dispensary to identify children who could benefit from stool testing. The STBCEU project team also supported the laboratory technicians who were processing the stool samples by monitoring laboratory results and reviewing data collected by laboratory technicians. The project team prepared monthly progress reports for each oblast based on the data received for discussion at monthly meetings. Based on these discussions, the team conducted mentoring visits to provide ad hoc onsite laboratory and clinical guidance as needed. Data of all children for whom stool was collected were compiled in a database and analyzed using descriptive statistics.

## 3. Results

During the pilot, which took place from 1 November 2021 to 30 September 2022, a total of 168 stool tests were conducted, with samples submitted in all 12 pilot regions. Table 1 shows the characteristics of the 168 children for whom stool was collected. By coincidence, equal numbers of male and female children submitted a stool sample and 34.5% of children were under 5 years of age. There were 9 stool samples collected from adolescents (over 15 years of age), ranging from 15 to 22 years. In total, 8 children (4.7%) were living with or had been exposed to HIV. For 123/168 children (73.2%), the preliminary TB diagnosis was based on chest X-ray with or without clinical signs and symptoms, and for 6 it was based solely on clinical diagnosis. For 30 children, the preliminary TB diagnosis was also based on laboratory findings. For 132/168 children (78.6%), in addition to stool, a respiratory sample also was collected, mainly gastric lavage (79 children) and nasopharyngeal wash (36 children). Classifying the method of collection as invasive or not, for 126/132 children (95.5%), the respiratory sample was obtained using more invasive methods and for just 6 children the respiratory sample was spontaneously expectorated.

Of the 168 stool samples tested, valid results were obtained for 162. Among these, 18 (11.1%) were MTB-positive; 9 MTB trace, 7 MTB detected low, and 2 MTB detected medium. In total, 6 (3.6%) samples had a final invalid/error Xpert test result. All of the 132 children with respiratory samples collected had valid Xpert test results, and 24/132 (18.2%) were MTB-positive; 8 MTB trace, 3 MTB detected very low, 12 MTB detected low, and 1 MTB detected medium. In addition to Xpert, culture results were available for 130 of the 132 respiratory samples (98.5%). There were 15 MTB-positive cultures, 111 MTB-negative cultures, and 2 contaminated cultures; for 2 samples, results were missing (in one case the sample was lost and in the other case the results were lost).

**Figure 2:**
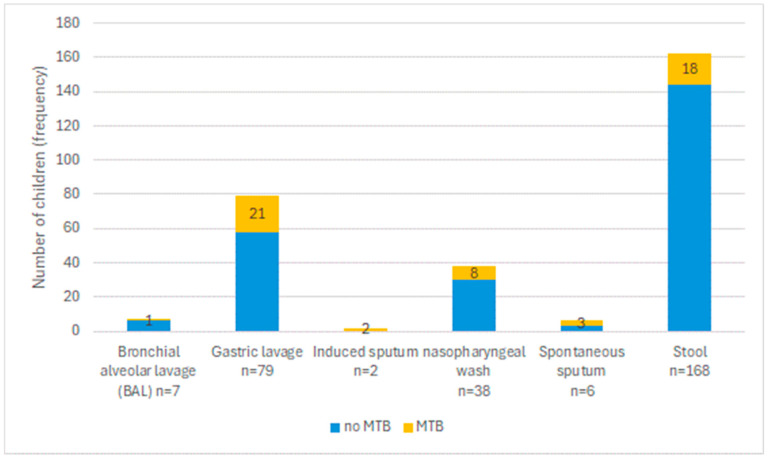
Collection of samples for tuberculosis (TB) diagnosis and *Mycobacterium tuberculosis* (MTB) positivity rate among children in pilot regions of Ukraine between November 2021 and September 2022.

Figure 2 shows the collection of different sample types including stool with their MTB positivity results. Not all children had all samples collected and tested. Stool, gastric lavage, and nasopharyngeal wash were the most collected sample types. MTB positivity varied among the different sample types but was over 10% in all sample types; because of the small numbers, differences were not assessed for significance.

**Figure 3:**
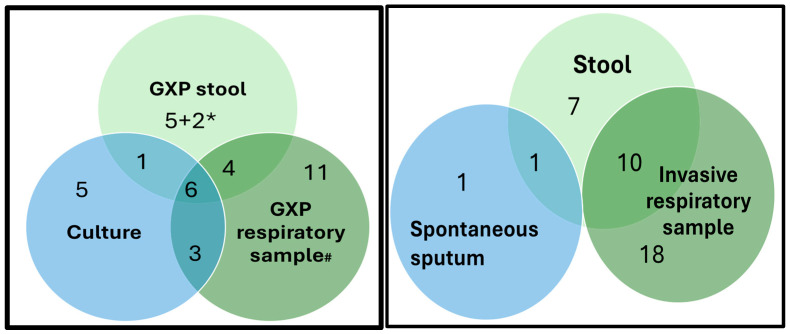
Venn diagram on *Mycobacterium tuberculosis* (MTB) positivity by type of test and sample type (**left**) and by invasiveness of sample collected (**right**) for the 37 MTB confirmed children. GXP = Gene Xpert MTB/RIF Ultra; * 2 samples did not have valid results on respiratory samples; # respiratory or other samples like gastric lavage, sputum, bronchial alveolar lavage, and nasopharyngeal wash.

Combining the MTB detection results from all samples and tests conducted indicated that of the 168 children, a valid MTB bacteriological result was available for 166 (98.8%). For two children for whom the only available sample was stool, the final test results were invalid/error. Overall, MTB was detected in 37 children; 22 based on Xpert only, 5 based on culture only, and 10 confirmed both on Xpert (stool or respiratory sample) and culture (Figure 3). In seven children, MTB was confirmed solely based on stool testing. Of these, five children had negative results on the respiratory sample (both culture and Xpert), and the other two children had no respiratory sample collected. In terms of invasiveness of the sample collected, of the 37 children with MTB detected, for 18 this was confirmed only in an invasive sample, for 10 in both an invasive and noninvasive (stool) sample, and for 9 children, MTB was only detected in a noninvasive sample (stool or spontaneous sputum), as shown in Figure 3.

Looking at concordance of all test results (MTB detected and MTB not detected) indicates that for the 128 children with a valid result on stool and a respiratory sample, results were concordant for 104/128 (81.3%), representing 11 MTB detected and 93 MTB not detected. Comparing just the Xpert results on stool and a respiratory sample showed a concordance of 108/128 (84.4%), representing 10 MTB detected and 98 MTB not detected. Concordance of MTB detection between stool and culture was 105/122 (86.1%), representing 7 MTB detected and 98 MTB not detected. Concordance between invasive and noninvasive samples was 100/122 (82.0%), representing 10 MTB detected and 90 MTB not detected among 122 children with valid results on both. For 18 children, MTB was only detected in the invasively obtained respiratory sample; 11 gastric lavage, 4 nasopharyngeal wash, 2 induced sputum, and 1 bronchial alveolar lavage. For 4 children, MTB was detected in stool but not the invasive respiratory sample. Details on MTB and rifampicin resistance detection for the different sample types and test assays for all 37 children with MTB detected during the pilot are outlined in the Supplementary Material (Table S1).

**Figure 4:**
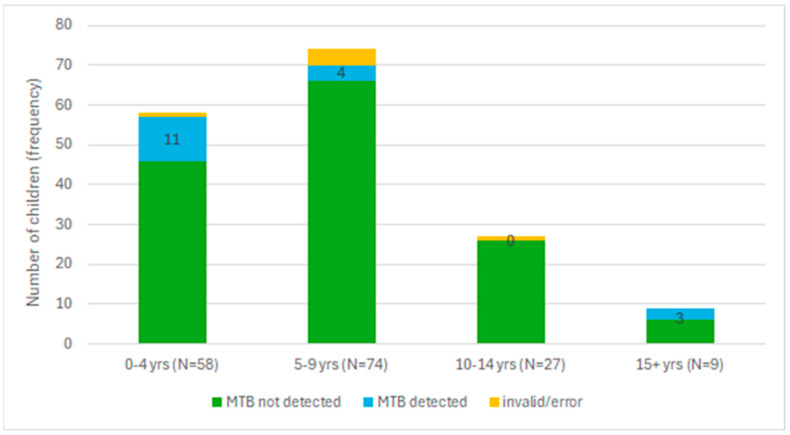
Number of stool samples collected and their results on Xpert MTB/RIF Ultra by age group.

Of the 18 children with MTB detected in stool, more than half (15/18) were below 10 years of age. As seen in Figure 4, children ages 0–4 years had a 19.0% MTB positivity rate (11 MTB-positive out of 58 total), children ages 5–9 years had a 5.4% MTB positivity rate (4/74), there were no MTB-positive children ages 10–14 years, and those 15 years and above had a 33.3% MTB positivity rate (3/9) in stool.

Among the 18 children with MTB detected in stool, 3 (16.7%) had rifampicin (Rif) resistance. In respiratory samples analyzed by Xpert, 3 out of 24 (12.5%) with MTB detected were Rif-resistant. These were not the same children and only one child was confirmed Rif-resistant in both stool and sputum. All 15 children with a culture positive result had drug susceptibility testing (DST) results and in 5 (33.3%), Rif resistance was confirmed. Combining the results on Rif resistance obtained by Xpert (on stool or respiratory sample) and culture shows that Rif resistance was detected in a total of seven children by one or multiple tests, and for one child, this was solely in stool (Table 2). For 15 children, sensitivity to rifampicin was confirmed in one or more samples. Thus, overall, in 7/22 (31.8%) participants with MTB detected who had Rif susceptibility results, Rif resistance was confirmed. For the other 15/37 children with MTB detected, a Rif susceptibility result was not available; for 13 children this was because the only valid bacteriological result for MTB was a trace detected, for which Rif status is indeterminate. The two other children had MTB detected low, but their Rif testing result was indeterminate on Xpert, and MTB was not detected by another test.

**Figure 5:**
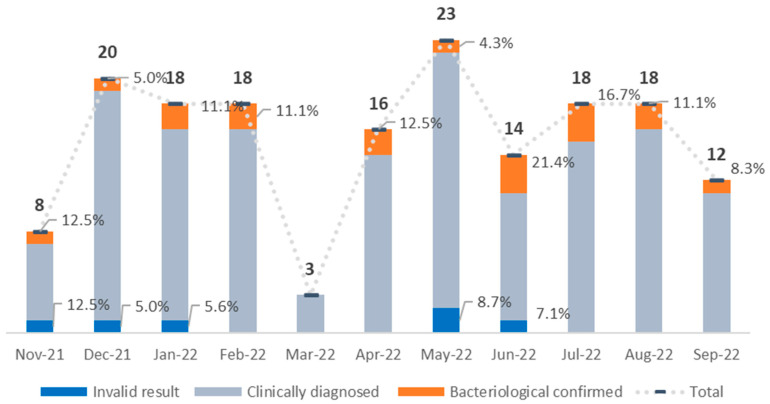
Number of stool tests performed in pilot regions, November 2021 to September 2022.

Figure 5 outlines the pattern of stool sample submission during the pilot, which started in November 2021. In the early period, from December 2021 to February 2022, close to 20 stool samples per month were submitted. In March 2022, during the first month of the war, stool testing came to an almost complete stop, but then resumed quickly back to 15–20 samples per month. Over the 11 months of implementation, 6 samples (3.6%) returned a final result of invalid or error, with 3 of these happening in the first months (November and January) and 3 mid-way (May and June).

The 168 children included all had a preliminary diagnosis of TB; after concluding all investigations and laboratory tests including on stool, 140 children were started on TB treatment. For the 28 children that did not start treatment, in 26 children TB was not confirmed, 1 child was lost to follow-up when they moved abroad at the start of the war, and relatives refused treatment for 1 child. Of the 140 children that started treatment, 130 children successfully completed treatment, 4 were lost to follow-up, 1 child had to stop treatment because of side effects, and 1 child died while on TB treatment, while for 4 children, no details on treatment outcome were available.

## 4. Discussion

Through advocacy, dedicated training, and on-site mentorship from the project team, using stool as a noninvasive sample was successfully introduced in 12 regions of Ukraine, despite the war. The pilot demonstrated that MTB can be detected in stool, as 18 of the 37 children with a bacteriological confirmation had MTB detected in their stool. For 11 of the 18, MTB was also detected in a respiratory sample which, for 10 children, was an invasive sample. Stool is a noninvasive sample and can contribute to obtaining a bacteriologically confirmed diagnosis of TB in children, including confirmation of rifampicin resistance [7].

Concordance of test results between stool and respiratory samples was high at 81%, and the concordance was slightly higher for culture than for Xpert. For 7 of the 18 children with bacteriological confirmation, stool was the sole sample wherein MTB was detected, suggesting stool can contribute to TB case finding in children (as has been shown in other settings [14,15]).

As has also been shown in other settings [14,16,17,18,19], testing stool could decrease the need for more invasive specimen collection methods, which are not available at all healthcare levels and are unpleasant for children and their parents/guardians. Using stool as an alternative sample is advantageous for children who cannot produce a spontaneous sputum. Of the 168 children with a stool sample included in this pilot, a respiratory sample was available for only 78% of them. Of the respiratory samples, just 6 children (5%) could provide a spontaneously produced sample, while for 126 children (95%), the respiratory sample was obtained via more invasive methods, including gastric lavage, nasopharyngeal wash, induced sputum, or bronchoalveolar lavage. Concordance of test results between invasive and noninvasive samples (stool or spontaneous sputum) was high at 82%.

Though young children more often have difficulty providing a spontaneous expectorated sputum sample. Older children and even adolescents who are not able to provide a spontaneously expectorated sample could also benefit from stool as an alternative non-invasive sample. As demonstrated in our pilot implementation, stool was collected for 9 adolescents (15–22 years) and MTB was detected in 3 of them, and for 2 this was in stool.

Using stool as an alternative sample may have a positive contribution to the ongoing efforts to decentralize TB care in Ukraine and bring diagnosis closer to people to improve treatment and care outcomes. Historically, TB care in Ukraine was centralized and only provided at central TB dispensaries in the oblasts. Since 2019, health systems reform has initiated the decentralization of services, making peripheral family doctors responsible for the detection, diagnosis, and treatment of TB. Rayons (administrative districts) were supplied with GeneXpert machines as a step in decentralization, so family doctors no longer need to send samples to a central facility and can process them locally.

This approach is part of the country’s broader strategy to improve treatment and care outcomes by making TB diagnosis more accessible and less invasive for children. Stool testing can be considered one of the tools in the effort to decentralize TB care. Patients can now consult with their family doctor, and if there is suspicion of pulmonary TB in cases where a child cannot produce sputum, a stool sample may be provided for bacteriological examination. This has the potential to improve treatment initiation and be cost-effective. A model-based analysis in Indonesia and Ethiopia found that collecting stool for Xpert testing at the primary healthcare level led to an 18% to 25% increase in pediatric TB treatment initiation and showed a high (>85%) probability of cost-effectiveness in both countries [20].

In addition to bacteriological confirmation of MTB, stool can also contribute to the identification and confirmation of resistance to rifampicin. This is especially relevant for high DR-TB burden countries like Ukraine, where a third of TB patients are estimated to have resistance to rifampicin [2]. In this pilot, seven children with Rif resistance were identified, a third of the children for whom results on Rif susceptibility status was available. For three children, Rif resistance was confirmed in stool and for one of them stool was the only source of Rif resistance confirmation.

While stool can help increase bacteriological confirmation and serve as an additional noninvasive sample, children often have paucibacillary disease and, therefore, a negative test, whether in stool or another sample. In the presence of other data supporting the diagnosis of TB, a negative stool test does not exclude a TB diagnosis.

Our analysis found that a significant proportion of samples with MTB detected had lower bacillary load; half of the samples detected a trace and 40% were MTB detected low. Trace is a confirmation of TB in a child and the child should be started on anti-TB treatment [7]. However, trace results do not provide a determinate result of rifampicin resistance. This highlights the importance of comprehensive diagnostic infrastructure parallel to introduction of stool testing. In high DR-TB burden countries, such as Ukraine, it is still essential to collect additional samples for culture and DST, and to collect information on the drug resistance pattern of the index patient to enable the prescription of the right treatment regimens. Anecdotal evidence indicates that Xpert XDR (extensively drug-resistant) can be performed on stool using the same SOS method, though for Ukraine this would not provide a sufficient profile of drug resistance. Since 2019, Ukraine has countrywide access to bedaquiline, linezolid, and delamanid—drugs that cannot be identified with Xpert XDR; therefore, surveillance for resistance to these drugs will still require DST.

The pilot results helped to guide the decision to implement stool as an alternative noninvasive sample in routine TB diagnosis in Ukraine. Following the pilot, from June 2022 onward, Ukraine’s national TB program began promoting the use of stool testing throughout the country. The results of the pilot helped to make the necessary changes to the TB diagnostic algorithm to incorporate stool as an alternative specimen for all children that cannot produce a spontaneous sputum sample as opposed to the more invasive techniques currently in use to obtain a bacteriological sample in such instances.

Stool-based GeneXpert testing is a feasible and valuable tool for TB diagnosis in children, capable of increasing bacteriological confirmation rates and identifying drug resistance. This method can enhance TB case finding and treatment initiation, contributing to better health outcomes for children in high TB burden settings. The use of stool as an additional sample for TB diagnosis can help reduce the need to subject children to gastric lavage or other invasive methods to collect a diagnostic sample. Bacteriological confirmation and early detection of drug resistance is especially important in a country like Ukraine with high levels of drug resistance. Early detection of TB and its drug-resistant strains in children will allow timely adjustments to provide safer and more effective treatment and save more lives.

## Figures and Tables

**Table 1:** Characteristics of children who were tested using stool samples.

Variables	Children Tested with Stool Samples (*n* = 168)
**Sex**	
Female	84 (50%)
Male	84 (50%)
**Age group**	
0–4 yrs.	58 (34.5%)
5–9 yrs.	74 (44%)
10–14 yrs.	27 (16%)
15+ yrs. †	9 (5.4%)
**HIV status**	
Negative/unknown *	160 (92.9%)
HIV+/exposed	8 (7.1%)
**Preliminary TB diagnosis**	
Clinically diagnosed	6 (3.6%)
Based on X-ray	122 (72.6%)
Clinical and laboratory diagnosis	5 (3.0%)
Clinical + X-ray	1 (0.6%)
Clinical + X-ray + laboratory	24 (14.3%)
Histopathology extrapulmonary #	1 (0.6%)
No information on preliminary TB diagnosis	9 (5.4%)
**Respiratory sample collection**	
Bronchial alveolar lavage	7 (4.2%)
Gastric lavage	79 (47.0%)
Induced sputum	2 (1.2%)
Nasopharyngeal wash	38 (22.6%)
Spontaneous sputum	6 (3.6%)
Not collected	36 (21.4%)

* It could not be confirmed whether all children were tested even though it is recommended per national guidelines. # This was an (isolated) case of extrapulmonary TB (sternal) with no other pulmonary sample collected; only a stool sample was collected; † Maximum age was 22 years.

**Table 2:** Details of test results for the 7 children with rifampicin resistance detected.

Child	Age	Sex	Xpert MTB/RIF Ultra Result, Other Sample ^#^	Xpert MTB/RIF Ultra Result, Stool Sample	Culture Result	DST Result
1	Under 1	M	MTB not detected	MTB not detected	Positive	Resistant
2	Under 1	M	MTB+ low concentration/RIF indeterminate	MTB+ low concentration/RIF+	Positive	Resistant
3	Under 1	F	MTB+ low concentration/RIF+	MTB not detected	Positive	Resistant
4	Under 1	F	MTB+ low concentration/RIF+	MTB+ low concentration/RIF+	Positive	Resistant
5	1	M	Not available	MTB+ low concentration/RIF+	Not available	Not available
6	9	M	MTB not detected	MTB not detected	Positive	Resistant
7	13	M	MTB + low concentration/RIF+	MTB not detected	Negative	Not applicable

^#^ Respiratory or other samples including gastric lavage, sputum, bronchial alveolar lavage, and nasopharyngeal wash. Abbreviations: DST, drug susceptibility testing; MTB, *Mycobacterium tuberculosis*; RIF, rifampicin.

## Data Availability

Dataset available on request from the authors, as long as the requester justifies the need for the data for a valid purpose; does not impede existing health programs; maintains public trust in data confidentiality; and avoids any conflict of interest.

## Supplementary Materials

The following supporting information can be downloaded at: https://www.mdpi.com/article/10.3390/tropicalmed9090209/s1, Table S1: Details on *Mycobacterium tuberculosis* (MTB) and rifampicin resistance detection for the different sample types and test assays for all 37 children with MTB detected during the pilot.
